# Identifying the patterns of ultra-processed food consumption and their characteristics in the UK adults using the UK National Diet and Nutritional Surveys 2008/09 to 2018/19

**DOI:** 10.1017/S1368980025100840

**Published:** 2025-08-22

**Authors:** Martino Bussa, Federico Ambrogi, Valeria Edefonti, Martin O’Flaherty, Yanaina Chavez-Ugalde, Zoè Colombet

**Affiliations:** 1 Bicocca Bioinformatics, Biostatistics and Bioimaging Centre, School of Medicine and Surgery, University of Milano-Bicocca, Milan, Italy; 2 Department of Clinical Sciences and Community Health, University of Milan – La Statale, Milan, Italy; 3 Fondazione IRCCS, Ca’ Granda Ospedale Maggiore Policlinico, Milan, Italy; 4 Department of Public Health, Policy and Systems, University of Liverpool, Liverpool, United Kingdom; 5 City St George’s, University of London, London, UK

**Keywords:** Ultra-processed food, Dietary pattern, Cluster analysis, Determinants

## Abstract

**Objective:**

To identify the dietary patterns of ultra-processed food (UPF) consumption in UK adults and to explore their nutritional characteristics and associated demographic and socio-economic factors.

**Design:**

UPF-based dietary patterns were identified using weighted principal component analysis and k-means cluster analysis on UPF intakes (identified using Nova classification) from the cross-sectional National Diet and Nutrition Survey data (2008–2019). Weighted multivariable logistic regression models were employed to identify the demographic and socio-economic factors associated with the patterns.

**Setting:**

United Kingdom.

**Participants:**

8347 adults (≥ 18 years).

**Results:**

UPF accounted for 54 % of total energy intake in the UK adult diet. Three distinct UPF-clusters were identified, labelled as ‘Sweet Foods’, ‘Fast Foods’ and ‘Traditional Foods’ based on their predominant food intakes. Older participants (> 68 years) were more likely to adhere to the ‘Sweet Foods’ pattern (OR: 2·39; 95 % CI: 1·99, 2·87) and less likely to be part of the ‘Fast Foods’ pattern (OR: 0·47; 95 % CI: 0·40, 0·55) compared with younger individuals (< 29). Participants in lower occupations were less likely to adhere to the ‘Fast Foods’ pattern than participants in the higher occupations (OR: 0·82; 95 % CI: 0·72, 0·94) while being more likely to adhere to the ‘Traditional Foods’ pattern (OR: 1·23; 95 % CI: 1·06, 1·43).

**Conclusions:**

The UK diet was dominated by UPF products. Our analysis identified three distinct UPF dietary patterns with varying nutritional quality, influenced by key demographic and social factors. These findings provide valuable insights into the determinants of UPF consumption and highlight which population groups are more likely to consume certain types of UPF.

The prevalence of overweight and obesity has been steadily increasing worldwide since 1980, posing a significant global challenge^(1)^. In 2021, two in three adults were either overweight or obese in England^(2)^. A large part of non-communicable disease deaths was attributable to poor diets, approximately 30% in 2019^(3)^, suggesting the improvement of diets as a well-established public health goal. The global rise in non-communicable diseases and obesity prevalence is partly attributed to rapid changes in global food systems, coupled with the development of increasingly sedentary lifestyles^(4)^. Recent social changes, such as technological evolution, urbanisation and increased per capita income, have profoundly influenced the global food system^(5)^, leading to the adoption of ‘Western’ dietary patterns. These patterns are characterised by high levels of saturated fats, sugars and refined foods and low intakes of fibre-rich foods, primarily due to the high availability of cheap, energy-dense, nutrient-poor foods^(6)^. Within this nutrition transition, increased intakes of ultra-processed foods (UPF) and ready-to-eat meals have been observed^(7)^. Nowadays, UPF have become the primary source of energy in most high-income countries: 48% of the total dietary energy intake in Canada^(8)^, 57·9 % in the USA^(9)^ and 56·8% in the UK^(10)^.

Multiple classifications exist to identify UPF, but the most frequently used is the Nova classification, which categorises all products into four progressively processed groups, based on the extent of processing, with the highest category labelled as UPF^(11)^. UPF are primarily industrial formulations made from substances extracted from foods, often chemically modified, and typically contain few ‘natural’ ingredients. Often, UPF contain a variety of cosmetic additives, including preservatives, stabilisers, emulsifiers, solvents, binders, bulking agents, sweeteners, thickeners, sensory enhancers, carbonating, flavours, flavour enhancers and colours^(11–13)^. UPF are often described as energy-dense, containing high levels of sugars, sodium and saturated fats, and low levels of fibres, micronutrients and phytochemicals^(14)^, even if considerable variation in these levels has been observed within each category of processing^(15)^. UPF play a substantial role in nutrient intake^(16)^, and a study published in 2023 demonstrated how healthy dietary patterns with a high diet quality score, and adequate amounts of most macro- and micronutrients can be achieved using UPF^(17)^. Indeed, the range of available UPF is broad, from pre-packaged whole meals or non-sugared flavoured water, as well as sugary soft drinks and highly processed snack foods like chips and candies.

While altering food from its natural state for safety, convenience, taste or palatability seems to have been a key contributor to human development, it also seems to be a substantial threat to health^(18)^. Especially, the consumption of UPF seems to be associated with cancer, CVD, type 2 diabetes and other non-communicable diseases^(19)^.

Given the significant role of UPF in modern diets and the growing evidence of their detrimental health effects, it is crucial to understand UPF consumption and its determinants to better inform effective public health policies. As UPF encompass a wide range of products, identifying patterns in their consumption – such as which types are consumed together – can shed light on their impact on health. Further, examining whether these patterns correlate with individual characteristics like age or socio-economic status can provide valuable insights for targeted interventions. Therefore, this study aims to identify the dietary patterns of UPF consumption among adults in the UK, exploring their nutritional characteristics and the associated demographic and socio-economic factors.

## Subjects and methods

### Data source

We conducted a secondary analysis of individual-level data from the UK National Diet and Nutrition Survey (NDNS) using waves 1–11 (2008/09–2018/19). Details of the rationale, design and methods of the survey have been described elsewhere. Briefly, the NDNS is an annual, cross-sectional survey collecting information on the nutrient intakes and status of individuals living in private households in the UK^(20–23)^. Between 2008 and 2019, the NDNS ran continuously as a rolling programme covering adults and children aged 1·5 years and over, using consistent data collection methods. A nationally representative sample was selected each year using a multi-stage random probability design, with one adult and one child chosen randomly from each household. Demographic and socio-economic data were collected through questionnaires, and anthropometric data were measured^(20–23)^. Dietary data were collected through 4-d food diaries administered on random days of the week to ensure a balanced representation of dietary intakes. Once completed, interviewers reviewed diaries with respondents to add any missing details and enhance completeness. All those who completed 3 or 4 d of dietary recording were included in the dataset, giving a sample size of 6828 participants for waves 1–4 combined, a sample size of 2546 participants for years 5–6 combined, a sample size of 2723 participants for waves 7–8 and a sample size of 3558 participants for waves 9–11.

### Inclusion criteria

Individuals who took part in NDNS waves 1–11, aged 18 years or older at data collection and who had completed at least three food diary days (for the 4-d food diary) were included in these analyses.

### Variables of interest

#### Socio-demographic characteristics

We used age in groups of 10 years, sex, country (England, Northern Ireland, Scotland and Wales) and ethnicity (white and non-white), as demographic variables, and occupational categories (higher occupations; intermediate occupations; lower occupations; small employers and own account workers; routine/semi-routine occupations; and never worked) as socio-economic variable in our analysis.

#### Ultra-processed food consumption

To identify the consumption of UPF in the NDNS dietary data, we used the Nova classification developed by Monteiro and colleagues^(11,13)^. According to the Nova classification, all foods and drinks can be classified into one of these four groups according to the extent and purpose of the industrial food processing they undergo: ‘minimally processed foods’, ‘processed culinary ingredients’, ‘processed foods’ and ‘ultra-processed foods’. Each food and drink in the NDNS data was classified by two independent researchers (ZC and YCU), leading to a final 97 % agreement after discussion, as detailed elsewhere^(23)^. For the present study, UPF intake was estimated using the percentage of daily total energy intake (without alcohol) provided by dietary items classified in the Nova group ‘ultra-processed foods’, while all other items (excluding alcohol) were classified as non-UPF.

### Statistical analysis

To identify the UPF dietary patterns, we used a two-step procedure, detailed in the Supplemental Material. First, a weighted principal component analysis (PCA) was applied to the covariance matrix of UPF intakes from twenty-one food groups (in % of energy intake per day). Then, a clustering procedure was applied to the first retained principal components (PC) to group participants based on similarity in UPF intakes operationalised via PCA, using a 50 % explained variance threshold. Cluster analysis was performed using the Hierarchical Clustering Partitional Clustering algorithm^(24)^, which combines hierarchical and partitional clustering. To determine the optimal number of clusters, we examined the dendrogram and various cluster validation indices starting from either the 4-PC or the 5-PC solutions. Given that the inclusion of the fifth PC did not enhance cluster separation or compactness, the first four PC were retained. PC loadings for the 4-PC solution were reported in online supplementary material, Supplemental Table 1 for completeness.

Cluster analysis identified distinct groups, interpreted as dietary patterns and labelled according to their predominant food intakes. Clusters were described according to their main nutritional characteristics. ANOVA was used to compare the means of nutritional characteristics (adjusted for daily energy intake) and food groups across the UPF-based dietary patterns; the Tukey test was used for pairwise comparisons. Weighted multivariable logistic regression models were employed to associate each cluster with selected demographic and socio-economic characteristics. Adjusted OR were reported with 95 % CI. A *P*-value of < 0·05 was considered statistically significant. Data management and statistical analyses were performed using R software, version 4.2.3, with the FactoMiner R package^(25)^.

## Results

Of the 15 655 participants across eleven survey waves, 8347 were adults (aged 18 and above), and all these adults had completed at least three food diary days. Consequently, 8347 participants were included in the analysis. The only missing data were for ethnicity (0·16 %) and occupation (0·53 %) (see online supplementary material, Supplemental Table 4). Overall, adults reported a mean total energy intake per day of 1761·73 kcal (sem 0·59), and 54 % of these calories were provided by UPF (Table 1). UPF contributed highly to carbohydrates, fats, free sugars and sodium intake.

**Table 1.** Daily intakes of selected nutritional characteristics in the adult population in the UK from the National Diet and Nutrition Survey 2009–2019 and associated WHO recommendations (*n* 8347)

	Total			UPF			Non-UPF			WHO recommendations
	Mean	sem	% of total energy	Mean	sem	% of total energy	Mean	sem	% of total energy	
Total intake (g/d)^*^	2699·80	5·61	–	671·32	2·07	–	2028·48	3·60		
Energy intake (kcal/d)	1761·73	0·59		947·98	0·73	53·81	813·75	0·89	46·19	
Carbohydrates (g/d)^*^	213·30	0·68	48·43	130·48	0·47	29·63	82·82	0·23	18·80	40–70 % of total energy intake
Total sugars (g/d)^*^	90·28	0·33	20·50	44·00	0·19	9·99	46·28	0·15	10·51	
Other sugars (g/d)^*^	1·96	0·01	0·45	1·28	0·004	0·29	0·68	0·002	0·16	
Free sugars (g/d)^*^	53·95	0·26	12·25	34·69	0·17	7·88	19·26	0·10	4·37	Less than 10 % of total energy intake^‡^
Proteins (g/d)^*^	71·73	0·19	16·29	30·92	0·10	7·02	40·81	0·10	9·27	The RDA of protein of 0·83 g per kg body weight per day^§^
Fats (g/d)^*^	66·23	0·25	33·83	37·12	0·15	18·96	29·11	0·10	14·87	Not exceed 30 % of total energy intake
SFA (g/d)^*^	24·69	0·09	12·61	12·73	0·05	6·50	11·96	0·04	6·11	Less than 10 % of total energy intake
Cis MUFA (g/d)^*^	24·16	0·09	12·34	14·28	0·06	7·30	9·88	0·03	5·04	15–20 % of total energy intake
Cis-*n*3 fatty acids (g/d)^*^	1·96	0·01	1·00	1·10	0·004	0·56	0·86	0·002	0·44	0·5–2 % of total energy intake
Cis-*n*6 fatty acids (g/d)^*^	9·34	0·03	4·77	5·98	0·02	3·05	3·36	0·01	1·72	2·5–9 % of total energy intake
Trans fatty acids (g/d)^*^	1·08	0·0004	0·55	0·46	0·002	0·23	0·62	0·002	0·32	Less than 1 % of total energy intake
	Total daily nutrient intake			UPF		% from UPF	Non-UPF		% from non-UPF	WHO
Cholesterol (mg/d)^*^	243·16	0·69		63·74	0·24	26·21	179·42	0·46	73·79	
AOAC fibres^†^ (g/d)^*^	18·09	0·04		9·90	0·03	54·73	8·19	0·02	45·27	30 g per day
Sodium (g/d)^*^	2·06	0·01		1·50	0·01	72·82	0·56	0·002	27·18	Less than 2 g per day
Water (g/d)^*^	2307·23	4·37		460·60	1·35	19·96	1846·63	3·03	80·04	

UPF, ultra-processed foods.

* Adjusted for daily total energy intake.

† AOAC fibre: Dietary fibre measured using methods from the Association of Official Analytical Chemists (AOAC).

‡ Ideally less than 5 % of total energy intake for additional health benefits.

§ For a healthy adult with minimal physical activity.

Cluster analysis allowed to identify three dietary patterns. These were labelled according to their intakes as ‘Sweet Foods’, ‘Fast Foods’ and ‘Traditional Foods’ and represented 23, 47 and 30 % of the sample, respectively.

### Differences in food groups and nutritional characteristics

Table 2 presents the UPF intakes (in % of daily energy) within the twenty-one food groups included in the PCA across the three identified dietary patterns. The largest cluster (3947 subjects), labelled the ‘Fast Foods’ pattern, included adults with, on average, the highest daily energy intake of UPF from pre-prepared meals, homemade dishes (e.g. meat pies and pastries), manufactured poultry, pizza, hamburgers and kebabs, chips, breakfast cereals and soft drinks compared with the other two clusters. The second cluster (1891 subjects), labelled the ‘Sweet Foods’ pattern, consisted of adults with the highest daily energy intake of UPF from biscuits and other sweet baked goods, industrial desserts and homemade desserts, as well as chocolate confectionery and yoghurts. Participants in this cluster had a lower daily energy intake of UPF from meat products, pizza, beans, chips, crisps and savoury snacks, sauces and gravies and notably, soft drinks, compared with the other clusters. The third cluster (2509 subjects), labelled the ‘Traditional Foods’ pattern, reported the highest average energy intake of UPF from bread, processed meat, beans, margarine and other spreads. Additionally, the intake of UPF from manufactured fish dishes, sugar confectionery, chocolate confectionery and yoghurts was lower in this cluster than in the other two.

**Table 2.** Daily intake of ultra-processed food (UPF) in the twenty-one food groups included in the principal component analysis across UPF dietary patterns and in the overall national sample of the adult population in the UK from the National Diet and Nutrition Survey 2009–19 (*n* 8347)

	All		Sweet foods		Fast foods		Traditional foods	
*n*			1891		3947		2509	
%			22·65		47·29		30·06	
	Mean	sem	Mean	sem	Mean	sem	Mean	sem
Intake of UPF in the food groups (% of energy/d)^*^								
Industrial desserts	1·46	0·003	1·81	0·065^a,b^	1·54	0·044^c^	1·05	0·056
Dessert homemade	0·46	0·001	0·53	0·036^b^	0·51	0·025^c^	0·33	0·031
Pre-prepared meals	3·70	0·003	3·34	0·122^a^	3·99	0·084^c^	3·53	0·105
Homemade dishes	2·26	0·002	2·15	0·077^a^	2·45	0·053^c^	2·03	0·067
Biscuits and other sweet baked goods	6·04	0·053	15·02	0·091^a,b^	3·14	0·063^c^	3·84	0·079
Manufactured poultry	1·38	0·002	1·12	0·075^a^	1·57	0·052^c^	1·28	0·065
Processed meat	2·81	0·005	2·24	0·086^a,b^	2·65	0·059^c^	3·47	0·074
Bread	11·33	0·055	9·00	0·101^a,b^	7·57	0·070^c^	18·98	0·087
Manufactured fish dishes	0·41	0·001	0·42	0·035	0·45	0·024^c^	0·33	0·031
Sauces, dressing and gravies	1·74	0·002	1·41	0·057^a,b^	1·86	0·039	1·79	0·049
Pizza	1·56	0·004	1·19	0·097^a^	1·93	0·067^c^	1·23	0·084
Hamburgers and kebabs	0·68	0·002	0·57	0·052^a^	0·82	0·035^c^	0·54	0·044
Beans	0·78	0·001	0·64	0·039^b^	0·74	0·027^c^	0·96	0·033
Breakfast cereals	3·38	0·005	3·40	0·093^a,b^	3·82	0·064^c^	2·68	0·080
Chocolate confectionary	2·03	0·004	2·37	0·081^b^	2·23	0·055^c^	1·48	0·069
Crisps and savoury snacks	1·75	0·001	1·58	0·066^b^	1·74	0·045	1·89	0·057
Soft drink	2·06	0·005	1·51	0·091^a^	2·55	0·062^c^	1·72	0·078
Sugar confectionary	0·33	0·001	0·40	0·033^b^	0·35	0·022^c^	0·24	0·028
Yoghourt	1·15	0·002	1·33	0·054^b^	1·28	0·037^c^	0·81	0·047
Margarine and other spreads	1·86	0·008	1·69	0·054^a,b^	1·24	0·037^c^	2·98	0·047
Chips	2·20	0·003	1·87	0·085^a^	2·50	0·059^c^	2·00	0·074

Values are presented as mean (standard error of the mean (sem)).

* Twenty-one food groups used in the weighted principal component analysis. All *P* values < 0·01.

*P* values were adjusted for multiple comparisons according to the false discovery rate method.

Contrasts (Tukey test): ^a^group Sweet Foods *v*. group Fast Foods *P* < 0·05; ^b^group Sweet Foods *v*. group Traditional Foods *P* < 0·05; ^c^group Fast Foods *v*. group Traditional Foods *P* < 0·05.

Table 3 describes the key nutritional characteristics of each UPF-based dietary pattern and provides an overview of the nutritional profile in the overall national sample study. Notably, the ‘Fast Foods’ pattern exhibited the lowest average carbohydrate content (118 g/d) and the lowest energy intake from UPF compared with the other two UPF patterns, with the lowest intake of fats and a low intake of protein. Conversely, the ‘Sweet Foods’ pattern showed higher daily intakes of total sugars from UPF, especially free sugars, and elevated levels of fats, saturated fats and cholesterol. Furthermore, the ‘Traditional Foods’ pattern showed the highest average sodium content from UPF (1719 mg/d) and the lowest intake from free sugars compared with the other two. Online supplementary material, Supplemental Table 2 provides daily percentages of energy intake from UPF in the food groups not included in the PCA across the identified UPF dietary patterns. Online supplementary material, Supplemental Table 3 details the daily intake (g/d) of selected food groups (included or not in the PCA) within the three identified dietary patterns.

**Table 3.** Selected nutritional characteristics across UPF dietary patterns and in the overall national sample of the adult population in the UK from the National Diet and Nutrition Survey 2009–2019 (*n* 8347)

	All		Sweet foods		Fast foods		Traditional foods	
*n*	8347		1891		3947		2509	
%			22·65		47·29		30·06	
	Mean	sem	Mean	sem	Mean	sem	Mean	sem
Total UPF intake (g/d)^*^	671·32	2·07	648·00	9·24^a^	679·00	6·34	677·00	7·98
Energy intake from UPF (kcal/d)	947·98	0·73	1057·00	9·68^a,b^	888·00	6·65^c^	961·00	8·34
Carbohydrates (g/d)^*^	130·48	0·48	143·00	0·97^a^	118·00	0·67^c^	141·00	0·84
Total sugar (g/d)^*^	44·00	0·19	52·70	0·61^a,b^	43·40	0·42^c^	38·40	0·53
Other sugar (g/d)^*^	1·28	0·004	1·49	0·04^a,b^	1·35	0·03^c^	1·03	0·04
Free sugar (g/d)^*^	34·69	0·17	41·70	0·57^a,b^	34·60	0·39^c^	29·50	0·50
Protein (g/d)^*^	30·92	0·10	30·10	0·25^b^	29·40	0·17^c^	34·00	0·22
Fat (g/d)^*^	37·12	0·15	40·90	0·31^a,b^	34·80	0·21^c^	37·90	0·27
SFA (g/d)^*^	12·73	0·05	15·20	0·12^a,b^	11·90	0·08^c^	12·30	0·11
Cis MUFA (g/d)^*^	14·28	0·06	15·30	0·14^a,b^	13·50	0·09^c^	14·70	0·12
Cis-*n*3 fatty acids (g/d)^*^	1·10	0·004	1·11	0·01^a,b^	1·03	0·01^c^	1·19	0·01
Cis-*n*6 fatty acids (g/d)^*^	5·98	0·02	6·03	0·06^a,b^	5·59	0·04^c^	6·55	0·05
Trans fatty acids (g/d)^*^	0·46	0·002	0·52	0·01^a,b^	0·42	0·01^c^	0·46	0·01
Cholesterol (mg/d)^*^	63·74	0·24	69·80	0·90^a,b^	62·00	0·62	62·00	0·77
AOAC^†^ fibre (g/d)^*^	9·90	0·03	10·24	0·09^a,b^	8·92	0·06^c^	11·20	0·08
Sodium (mg/d)^*^	1503·22	4·92	1441·00	12·41^a,b^	1396·00	8·52^c^	1719·00	10·72
Water (g/d)^*^	460·60	1·35	422·00	8·52^a,b^	485·00	5·85^c^	451·00	7·36

UPF, ultra-processed food.

Values are presented as mean (standard error of mean (sem)).

* Adjusted for total daily energy intake.

† AOAC fibre: Dietary fibre measured using methods from the Association of Official Analytical Chemists (AOAC).

All *P* values < 0·01; *P* values were adjusted for multiple comparisons according to the false discovery rate method.

Contrasts (Tukey test): ^a^group Sweet Foods *v*. group Fast Foods *P* < 0·05; ^b^group Sweet Foods *v*. group Traditional Foods *P* < 0·05; ^c^group Fast Foods *v*. group Traditional Foods *P* < 0·05.

### Differences in demographic and socio-economic characteristics

Online supplementary material, Supplemental Table 4 describes the demographic and socio-economic characteristics of the participants and their distribution across the identified UPF dietary patterns. In our sample, 59 % of participants were females, 60 % lived in England, 92 % were Whites, 15 % were engaged in higher occupations, 33 % in lower occupations, 26 % in semi-routine/routine occupations and 11 % were small employers and self-employed workers. The sample exhibited a balanced distribution across all age groups.

Associations between each UPF dietary pattern and demographic/socio-economic characteristics are presented in Table 4 as OR and corresponding 95 % CI. Older participants (age > 68 years) were more likely to adhere to the ‘Sweet Foods’ pattern (OR: 2·39; 95 % CI: 1·99, 2·87) and less likely to adhere to the ‘Fast Foods’ pattern (OR: 0·47; 95 % CI: 0·40, 0·55) compared with younger individuals (age < 29 years). Women were also more likely to adhere to the ‘Sweet Foods’ patterns than men (OR: 1·40; 95 % CI: 1·26, 1·56), while being less likely to adhere to the ‘Traditional Foods’ (OR: 0·75; 95 % CI: 0·69, 0·83). Regarding the country, participants residing in Northern Ireland were more likely to adhere to the ‘Sweet Foods’ (OR: 1·28; 95 % CI: 1·10, 1·50) and less likely to adhere to the ‘Fast Foods’ pattern (OR: 0·76; 95 % CI: 0·67, 0·88) compared with those in England. Similarly, in Scotland, participants were less likely to adhere to the ‘Fast Foods’ pattern (OR: 0·84; 95 % CI: 0·74, 0·96) than in England.

**Table 4.** Associations between demographic and socio-economic characteristics and ultra-processed food dietary patterns in a sample of the adult population in the UK from the National Diet and Nutrition Survey 2009–2019 (*n* 8347)^*^

	Sweet foods *v*. other patterns			Fast foods *v*. other patterns			Traditional foods *v*. other patterns		
	OR	95 % CI	*P*-value^†^	OR	95 % CI	*P*-value^†^	OR	95 % CI	*P*-value^†^
Age (years)									
18–28	Ref.			Ref.			Ref.		
29–38	1·05	0·86, 1·29	0·690	0·85	0·73, 0·99	0·056	1·18	1·0, 1·40	0·108
39–48	1·29	1·07, 1·56	**0·020**	0·77	0·66, 0·89	**0·001**	1·14	0·96, 1·34	0·182
49–58	1·29	1·06, 1·56	**0·020**	0·82	0·71, 0·96	**0·022**	1·05	0·89, 1·24	0·589
59–68	1·30	1·06, 1·58	**0·020**	0·74	0·64, 0·87	**0·001**	1·17	0·99, 1·39	0·120
> 68	2·39	1·99, 2·87	**< 0·001**	0·47	0·40, 0·55	**< 0·001**	1·13	0·96, 1·33	0·200
Sex									
Men	Ref.			Ref.			Ref.		
Women	1·40	1·26, 1·56	**< 0·001**	1·01	0·93, 1·11	0·791	0·75	0·69, 0·83	**< 0·001**
Country									
England	Ref.			Ref.			Ref.		
Northern Ireland	1·28	1·10, 1·50	**0·008**	0·76	0·67, 0·88	**0·0004**	1·11	0·96, 1·28	0·202
Scotland	1·17	1·01, 1·37	0·076	0·84	0·74, 0·96	0·022	1·07	0·92, 1·23	0·413
Wales	0·77	0·65, 0·91	**0·009**	0·98	0·86, 1·12	0·791	1·24	1·08, 1·43	**0·010**
Ethnicity									
White	Ref.			Ref.			Ref.		
Non-white	0·99	0·81, 1·22	0·954	0·92	0·78, 1·09	0·442	1·10	0·92, 1·31	0·341
Occupational categories									
Higher occupations	Ref.			Ref.			Ref.		
Intermediate occupation	0·90	0·73, 1·12	0·417	0·93	0·78, 1·11	0·501	1·19	0·97—1·45	0·142
Lower occupation	1·04	0·88, 1·22	0·700	0·82	0·72, 0·94	**0·011**	1·23	1·06, 1·43	**0·024**
Small employers and own account workers	0·87	0·71, 1·08	0·279	0·92	0·77, 1·09	0·442	1·24	1·03, 1·50	0·061
Semiroutine/routine occupation	0·89	0·75, 1·05	0·244	0·75	0·65, 0·86	**0·00024**	1·56	1·33, 1·82	**< 0·001**
Never worked and other	0·78	0·57, 1·06	0·173	0·89	0·70, 1·14	0·442	1·40	1·08, 1·82	**0·033**

* Weighted multivariable logistic regression models adjusted for age, sex, country, ethnicity and occupational categories.

† *P* values were adjusted for multiple comparisons according to the false discovery rate method.

Values in bold indicate statistical significance at p < 0.05.

Regarding occupation, few associations were observed, but participants in lower and semi-routine/routine occupation categories were less likely to adhere to the ‘Fast Foods’ pattern than participants in the higher occupation category (OR: 0·82; 95 % CI: 0·72, 0·94 and OR: 0·75; 95 % CI: 0·65, 0·86, respectively), while being more likely to adhere to the ‘Traditional Foods’ pattern (OR: 1·23; 95 % CI: 1·06, 1·43 and OR: 1·56; 95 % IC: 1·33, 1·82, respectively). Interestingly, the participants who never worked and the retired ones were more likely to adhere to the ‘Traditional Foods’ pattern than participants in the higher occupations category (OR: 1·40; 95 % CI: 1·08, 1·82).

## Discussion

This study represents the first attempt to identify and describe dietary patterns of UPF intake among the adult population in the UK. Using a large, nationally representative sample, we identified three main UPF dietary patterns based on the type of ultra-processed products consumed: ‘Fast Foods’, ‘Sweet Foods’ and ‘Traditional Foods’, which differ by their nutritional intakes and the individual characteristics of their members.

In our sample, over half of the daily calories (54 %) consumed by the UK adult population between 2008 and 2019 came from UPF. These findings are consistent with previous analyses of the same NDNS data and other studies conducted in different countries^(9,15,26)^. For instance, Rauber and colleagues^(10)^ found that UPF contributed to 56·8 % of total energy intake and 64·7 % of total free sugars in the UK diet based on NDNS 2008–2014 data. Additionally, in the NDNS 2008–2012, an average of 53 % of total energy intake was provided by UPF, while minimally processed foods contributed to an average of 28 %^(18)^. Moreover, household food purchase surveys conducted in 2008 revealed that UPF constituted 63 % of energy in the UK^(12)^.

But the number of products that fall into the category of UPF according to the Nova classification system is high, and the type of ultra-processed products is broad. Within our sample, we have identified three UPF dietary patterns based on the main type of UPF consumed. Almost half of the adults in our sample tend to eat UPF we can classify as fast foods (e.g. prepared meals, pizza, hamburgers and kebabs, from the ‘Fast Foods’ pattern). In contrast, one in two adults ate traditional UPF (e.g. bread, processed meat, beans, margarine and other spreads, from the ‘Traditional Foods’ pattern). Finally, the other adults tend to eat ‘sweet’ UPF, such as biscuits, desserts, chocolate confectionery and yoghurts, from the ‘Sweet Foods’ pattern.

The high consumption of UPF observed in the UK adult diet raises important nutritional concerns. Indeed, the diet observed in our sample exceeded recommended intake levels for free sugars, saturated fat and sodium, while fibre intake fell below the recommended levels, according to WHO recommendations^(27–30)^. And UPF seem to highly contribute to these unhealthy nutritional intakes. This is consistent with previous studies, which have found high salt, free sugar and fat intakes associated with UPF^(8,10,15)^. UPF products accounted for more than 70 % of the daily sodium intake in our sample and 64 % of the daily free sugar intake. UPF foods had almost 30 % more fat than the diet fraction made up of non-UPF foods.

Interestingly, all the dietary patterns identified in our sample exhibited elevated fats, free sugars and sodium levels, even if they varied in nutritional content. Specifically, the ‘Sweet Foods’ patterns demonstrated significantly higher levels of fats, free sugars and energy content than the other two. In contrast, the ‘Traditional Foods’ dietary pattern exhibited the highest daily sodium intake. These findings corroborate existing research indicating that the specific types of UPF consumed can significantly impact overall nutritional intake. Some UPF may offer a comparatively healthier nutritional profile than others, as previously demonstrated^(17)^. However, it is important to note that diets heavily reliant on UPF, regardless of individual product choices, are still contributing to a high consumption of fat, salt, sugar and energy, which can be detrimental to health.

An interesting finding is the variation in UPF dietary patterns among adults in the UK, based on age, sex and occupation. Our results highlighted that older individuals were more likely to consume sweet UPF and fewer ready-to-eat foods (‘Fast foods’) than younger individuals. This variation may be attributed to age-related declines in taste and smell sensitivity^(31–33)^, as well as a slower perception of sweetness in older compared with younger adults^(34)^. These changes in sweet taste perception can influence food preferences^(35)^. Additionally, for older adults, particularly those who are homebound, factors such as sensory appeal (or aesthetic appeal), convenience and price were identified as playing a crucial role in food selection^(36,37)^. Conversely, younger participants showed a preference for ‘Fast Food’ UPF, a trend consistent with other national surveys that identified the youngest adult age group as having the highest consumption of junk foods^(18,26,38–40)^. Early adulthood is a period of life transition during which changes such as leaving the parental home or moving from school to further education or paid employment can lead to the disruption of pre-existing eating habits and dietary behaviours. Notably, leaving home and finishing school are associated with negative dietary changes^(41)^. These transitions may present critical opportunities for implementing effective interventions targeting diet and obesity. Similarly, women exhibited a preference for sweet UPF compared with men, which is coherent with the literature, for example, study showing women having a strong liking for the fat-and-sweet sensation^(42)^, study reporting more craving for sweet foods^(43)^ or reporting that women find sweet foods more pleasant^(44)^.

Finally, occupation is also a determinant of food choice, and its association with higher UPF intake varies between countries^(45)^. In our study, we observed an association between the type of UPF chosen and occupation, with adults in low, routine and semi-routine occupations showing a reduced preference for the ‘Fast Foods’ UPF pattern compared with those in higher-level occupations, instead favouring the ‘Traditional Foods’ UPF pattern. These findings underline the importance of considering age, gender and occupation differences when formulating dietary policies to promote healthier and more sustainable dietary choices, as well as understanding how other factors, such as marketing or price, influence choice in these groups.

Our study has some strengths and limitations. One key strength is the use of data from the NDNS, a large and nationally representative sample of the UK adult population, applying weighting to reduce sampling and non-response bias. Additionally, food diaries were used to collect dietary data, which are among the most comprehensive methods for assessing dietary intake^(10)^. Food diaries provide detailed accounts of consumed foods and are less prone to recall bias compared with other dietary assessment tools. However, there are also limitations to consider. The dietary data were self-reported, which may introduce self-reporting bias, including the under-reporting of certain items (notably, unhealthy foods). There is also a potential for misclassification into the Nova classification due to the lack of detailed ingredient information in some foods in NDNS. Furthermore, multivariate methods come with inherent limitations, as they require subjective decisions regarding the number of components extracted, the choice of clusters and their interpretation and naming. Such subjectivity can impact both the patterns identified and their subsequent interpretation, a limitation commonly acknowledged in studies employing these techniques^(46,47)^. It is also important to note that this is a cross-sectional study, so causal effects cannot be inferred. Also, as UPF consumption seems to have increased between the study period (2008–2018) and is likely to have increased since 2018, it will be interesting to evaluate if the UPF dietary patterns are changing with time. Additionally, the data were collected before the COVID-19 pandemic and recent inflation, a period that may have significantly altered dietary patterns in the UK.

Whether UPF-based diets are harmful to health simply due to poor nutritional quality, or if the nature and extent of the processing itself have health consequences, remains an ongoing debate that warrants continued research^(19,48,49)^. Based solely on their nutritional qualities, however, the three identified UPF patterns do not provide a sufficient causal basis to inform food policies. They do, however, provide good evidence on dietary patterns and socio-demographic and economic determinants of UPF consumption. Our findings suggest that policy efforts should focus on reducing overall UPF intake, while also improving the relative nutritional quality of UPF – for instance, through reformulation – and promoting the intake of fresh and minimally processed foods.

### Conclusion

This study confirms the continued dominance of UPF in UK diets. Our analysis identified three distinct dietary patterns with varying nutritional quality, shaped by key demographic and socio-economic factors. These findings provide valuable insights into the determinants of UPF consumption and highlight which population groups are more likely to consume certain types of UPF.

## Supporting information

Bussa et al. supplementary material Bussa et al. supplementary material

## Supplementary material

For supplementary material accompanying this paper, visit https://doi.org/10.1017/S1368980025100840.
