# Supplementary material for: Identifying the patterns of ultra-processed food consumption and their characteristics in the UK adults using the UK National Diet and Nutritional Surveys 2008/09 to 2018/19
Source: Public Health Nutr. 2025 Aug 22;28(1):e149. doi: 10.1017/S1368980025100840 (PMC12516623; doi:10.1017/S1368980025100840)
Supplement: Bussa et al. supplementary material [file S1368980025100840sup001.docx]

**Identifying the patterns of ultra-processed food consumption and their characteristics in the UK adults using the UK National Diet and Nutritional Surveys 2008/09 to 2018/19**

**Detailed statistical analysis**

To identify the dietary patterns in UPF intakes, we used a two-step procedure. Firstly, a weighted principal component analysis (PCA) was applied to 21 food group intakes containing the UPFs as a percentage of energy intake per day, using the covariance matrix. PCA generates independent linear combinations of the initial food-group variables, maximizing the explained variance^(1)^. We performed a weighted PCA using study weights provided by NDNS, which account for any biases introduced during household and individual selection, as well as address non-response bias at the individual level^2^. To ensure comparability, the weights were rescaled since the number of participants varied across datasets/years and were adjusted for the adult subsample, following the guidance provided in the NDNS study weights documentation^(2,3)^. The selection of the number of principal components was performed based on the following criteria: scree test, cumulative variance percentage, and a consideration of how the separation and compactness of the further identified clusters varied with different numbers of principal components; this left us with two possible solutions including four or five principal components. Then, to identify and group homogeneous elements within the dataset we performed a clustering procedure on the first retained principal components. To determine the optimal number of clusters, we examined the dendrogram and utilized the NbClust function from the NbCluster R package, choosing Euclidean distance as the similarity metric, and the ward.d2 as the clustering method^(4)^. Utilizing the first four principal components, various cluster validation indices were examined, including the Calinski and Harabasz index and the Hartigan index. Out of 23 calculated cluster validation indices, eight indicated that the optimal number of clusters was 3. Sensitivity analysis using five principal components still suggested an optimal number of 3 clusters. But the k-means analysis using four principal components provided better data separation than k-means with five principal components. Given that the inclusion of the fifth principal component did not improve the clustering analysis, as it did not result in enhanced separation or cluster compactness, the first four principal components were retained (Factor Loadings available in Supplementary Table 1). This decision represented a balanced compromise, achieving effective separation between elements into distinct clusters while preserving the cohesion of the clusters. Cluster analysis was performed using the HCPC algorithm implemented through the HCPC function in the FactoMiner R package^(5)^. The HCPC function in R combines hierarchical clustering with partitional clustering. It initially establishes the partition based on the hierarchical tree and then refines the results through iterative applications of the k-means algorithm. The Ward's hierarchical classification of individuals was applied to the four-dimensional space defined by the retained principal components.

Cluster analysis identified distinct groups, interpreted as dietary patterns and labelled according to their predominant food intakes. Clusters were described according to their main nutritional characteristics. UPF nutrient intakes were adjusted for daily energy intake. ANOVA was used to compare the means of nutritional characteristics and food groups across the UPF-based dietary patterns, and the Tukey test was used for pairwise comparisons. Weighted multivariable logistic regression models were employed to determine the strength of the association between each cluster and demographic and socioeconomic characteristics. Adjusted odds ratios (ORs) were reported with 95% confidence intervals (CIs). A p-value of < 0.05 was considered statistically significant. Data management and statistical analyses were performed using the R software, version 4.2.3.

**Supplemental Table 1.** Principal components loadings for the first four components from principal component analysis in the overall sample of the adult population in the UK from NDNS 2009-19 (n= 8,347)

|  | PCA1  (% expl.^a^ = 18.12) | PCA2  (% expl.^a^ = 13.86) | PCA3  (% expl.^a^ = 10.37) | PCA4  (% expl.^a^ = 7.77) |
| --- | --- | --- | --- | --- |
| Industrial desserts | -0.053 | 0.020 | -0.020 | -0.042 |
| Desserts homemade | -0.012 | 0.001 | -0.019 | -0.023 |
| Pre-prepared meals | -0.024 | -0.119 | 0.975 | -0.132 |
| Homemade dishes | -0.016 | -0.036 | -0.027 | -0.052 |
| Biscuits and other sweet baked goods | -0.453 | 0.864 | 0.113 | 0.137 |
| Manufactured poultry | -0.002 | -0.048 | 0.065 | 0.152 |
| Processed meat | 0.084 | -0.025 | 0.052 | 0.069 |
| Bread | 0.867 | 0.433 | 0.062 | 0.002 |
| Manufactured fish dishes | -0.009 | -0.000 | -0.011 | -0.031 |
| Pizza | 0.002 | -0.092 | -0.023 | 0.513 |
| Hamburgers and kebabs | -0.000 | -0.032 | 0.010 | 0.106 |
| Sauces, dressing and gravies | 0.017 | -0.029 | -0.038 | 0.018 |
| Margarine and other spread | 0.123 | 0.080 | 0.046 | -0.004 |
| Beans | 0.021 | -0.002 | 0.008 | 0.000 |
| Chips | 0.008 | -0.090 | 0.045 | 0.317 |
| Crips and savoury snaks | 0.024 | -0.020 | 0.042 | 0.166 |
| Soft drinks | -0.008 | -0.135 | 0.077 | 0.502 |
| Breakfast cereals | -0.100 | -0.053 | -0.088 | -0.493 |
| Chocolate Confectionery | -0.055 | -0.014 | 0.011 | 0.151 |
| Yoghourt | -0.043 | -0.000 | -0.033 | -0.092 |
| Sugar confectionary | -0.011 | 0.000 | -0.002 | 0.021 |

^a^Proportion on the variance explained by each principal component

**Supplemental Table 2**. Daily intake of UPF in food groups non included in the principal component analysis across UPF dietary patterns and in the overall national sample of the adult population in the UK from NDNS 2009-19 (n=8347)

|  | All | Sweet Foods | Fast Foods | Traditional Foods |
| --- | --- | --- | --- | --- |
| Size n (%) | 8347 | 1891 (22.65) | 3947 (47.29) | 2509 (30.6) |
| Intake of UPF in the food groups  (% of energy/day) |  |  |  |  |
| Cheese | 0.37 ± 0.0002 | 0.40 ± 0.03 | 0.36 ± 0.02 | 0.37 ± 0.03 |
| Dairy alternatives | 0.01 ± 0.0004 | 0.10 ± 0.02 | 0.13 ± 0.01^c^ | 0.05 ± 0.01 |
| Fruits | 0.27 ± 0.0003 | 0.31 ± 0.03 | 0.28 ± 0.02 | 0.24 ± 0.03 |
| Manufactured vegetable dishes | 0.17 ± 0.0001 | 0.15 ± 0.02 | 0.18 ± 0.01 | 0.16 ± 0.02 |
| Manufactured soups | 0.51 ± 0.0005 | 0.51 ± 0.03 | 0.47 ± 0.02^c^ | 0.57 ± 0.03 |
| Meat alternatives | 0.16 ± 0.0001 | 0.16 ± 0.02 | 0.17 ± 0.02 | 0.16 ± 0.02 |
| Nuts and seed | 0.20 ± 0.0004 | 0.18 ± 0.03 | 0.18 ± 0.02^c^ | 0.26 ± 0.02 |
| Other cereals | 0.44 ± 0.001 | 0.35 ± 0.04^a^ | 0.52 ± 0.03^c^ | 0.38 ± 0.04 |
| Other dairy | 0.32 ± 0.0003 | 0.32 ± 0.03 | 0.35 ± 0.02 | 0.28 ± 0.03 |
| Potatoes and tubercules | 0.24 ± 0.00001 | 0.23 ± 0.02 | 0.24 ± 0.02 | 0.24 ± 0.02 |
| Preserve | 0.31 ± 0.001 | 0.43 ± 0.02^a^ | 0.17 ± 0.01^c^ | 0.43 ± 0.02 |
| Vegetables | 0.36 ± 0.0003 | 0.35 ± 0.03 | 0.39 ± 0.02 | 0.33 ± 0.02 |

Vales are presented as mean ± standard error of mean

P values were adjusted for multiple comparisons according to the False Discovery Rate method (FDR)

Contrasts (Tukey test):

a: group Sweet Foods vs group Fast Foods p <0.05;

c: group Sweet Foods vs group Traditional Foods p <0.05;

**Supplemental Table 3**. Daily intake of selected food groups across UPF dietary patterns and in the overall national sample of the adult population in the UK from NDNS 2009-19 (n=8347)

|  | All | Sweet Foods | Fast Foods | Traditional Foods |
| --- | --- | --- | --- | --- |
| Size n (%) | 8347 | 1891 (22.65) | 3947 (47.29) | 2509 (30.06) |
|  | Mean ± SD | Mean ± SD | Mean ± SD | Mean ± SD |
| Energy intake (kcal/day) | 1807.08 ± 566.19 | 1869.39 ± 546.18 | 1814.88 ± 583.43 | 1748.89 ± 545.35 |
| Food groups (g/day) |  |  |  |  |
| Pasta, rice and other cereals | 80.97 ± 93.46 | 73.47 ± 93.49 | 93.28 ± 99.87 | 65.93 ± 78.42 |
| Vegetables^1^ | 138.80 ± 111.99 | 134.92 ± 107.00 | 144.80 ± 114.53 | 131.64 ± 110.72 |
| Potatoes^2^ | 81.02 ± 66.90 | 79.88 ± 65.95 | 83.43 ± 69.00 | 77.85 ± 63.83 |
| Eggs and eggs dishes | 25.50 ± 35.02 | 20.68 ± 29.79 | 25.25 ± 39.66 | 22.64 ± 29.73 |
| Cheeses^3^ | 30.03 ± 41.08 | 31.19 ± 40.36 | 29.39 ± 42.33 | 30.25 ± 39.44 |
| Milk^4^ | 152.55 ± 137.80 | 168.24 ± 141.71 | 158.24 ± 147.39 | 131.71 ± 113.91 |
| Yoghurts | 29.93 ± 51.70 | 35.06 ± 51.92 | 32.51 ± 55.50 | 21.09 ± 43.41 |
| Fruit | 99.90 ± 107.40 | 104.00 ± 99.65 | 106.40 ± 114.42 | 86.10 ± 99.17 |

Values are presented as mean and standard deviation

^1^salad and other raw vegetables, and vegetables not raw

^2^chips, fried roast potatoes, and other potato and potato salad dishes

^3^cheddar cheese, cottage cheese, and other cheeses

^4^ high-fibre breakfast cereals and other breakfast cereals

^5^ whole milk, semi-skimmed milk, skimmed milk, and other milk and cream

|  | All (%) | Sweet Foods (%) | Fast Foods (%) | Traditional Foods (%) |
| --- | --- | --- | --- | --- |
| **Sex** |  |  |  |  |
| Men | 41.91 | 35.94 | 41.99 | 46.23 |
| Women | 58.09 | 64.06 | 58.01 | 53.77 |
| **Age class** (in years) |  |  |  |  |
| 18-28 | 16.17 | 12.46 | 18.44 | 15.35 |
| 29-38 | 15.91 | 13.17 | 16.89 | 16.41 |
| 39-48 | 18.58 | 18.03 | 18.79 | 18.66 |
| 49-58 | 17.12 | 16.42 | 17.99 | 16.27 |
| 59-68 | 14.99 | 14.28 | 14.91 | 15.63 |
| >68 | 17.23 | 25.64 | 12.98 | 17.68 |
| **Country** |  |  |  |  |
| England | 60.88 | 59.68 | 62.87 | 58.64 |
| Northen Ireland | 12.85 | 14.69 | 11.57 | 13.50 |
| Scotland | 13.36 | 14.91 | 12.60 | 13.41 |
| Wales | 12.91 | 10.72 | 12.96 | 14.45 |
| **Ethnicity** |  |  |  |  |
| White | 91.89 | 92.92 | 91.57 | 91.61 |
| Non-white | 7.95 | 6.98 | 8.23 | 8.22 |
| NA | 0.16 | 0.10 | 0.20 | 0.17 |
| **Occupational categories** |  |  |  |  |
| Higher occupations | 15.21 | 15.24 | 16.65 | 12.93 |
| Intermediate occupations | 9.70 | 9.55 | 10.17 | 9.07 |
| Lower occupations | 33.12 | 34.82 | 32.74 | 32.44 |
| Semiroutine/routine occupations | 26.19 | 25.90 | 24.00 | 29.87 |
| Small employers and own account workers | 11.06 | 10.40 | 11.50 | 10.85 |
| Never worked and other | 4.18 | 3.57 | 4.37 | 4.34 |
| NA | 0.54 | 0.52 | 0.57 | 0.50 |

**Supplemental Table 4** Demographic and socioeconomic characteristics across UPF dietary patterns and the overall sample of the adult population in the UK from NDNS 2009-19 (n=8347)

References

1. Gleason PM, Boushey CJ, Harris JE, et al. (2015) Publishing nutrition research: a review of multivariate techniques--part 3: data reduction methods. *J Acad Nutr Diet* **115**, 1072–1082.

2. Adams J & White M (2015) Characterisation of UK diets according to degree of food processing and associations with socio-demographics and obesity: cross-sectional analysis of UK National Diet and Nutrition Survey (2008–12). *International Journal of Behavioral Nutrition and Physical Activity* **12**, 160.

3. University of Cambridge & NatCen Social Research (2019) *National Diet and Nutrition Survey*. UK Data Service.

4. Charrad M, Ghazzali N, Boiteau V, et al. (2014) NbClust: An R Package for Determining the Relevant Number of Clusters in a Data Set. *Journal of Statistical Software* **61**, 1–36.

5. Lê S, Josse J & Husson F (2008) FactoMineR: An R Package for Multivariate Analysis. *Journal of Statistical Software* **25**, 1–18.
